# Retraction of “N_2_/H_2_ Plasma-Enhanced Atomic Layer Deposition for SiO_2_ Gap Filling: Implications for Nanoelectronics in Semiconductor Manufacturing”

**DOI:** 10.1021/acsanm.5c05590

**Published:** 2025-12-31

**Authors:** Sameh Okasha, Donald Munson, Jun Yoshikawa

The editor retracts this article (DOI: 10.1021/acsanm.4c06844) following investigations and requests from ASM International due to an allegation that the article contained proprietary and confidential information. As such, the article has been taken down and is no longer available.

The original article was published on March 6, 2025 and retracted on December 31, 2025.

